# Shall We Tune? From Core-Shell to Cloud Type Nanostructures in Heparin/Silica Hybrids

**DOI:** 10.3390/polym14173568

**Published:** 2022-08-30

**Authors:** Giulio Pota, Giuseppe Vitiello, Virginia Venezia, Francesca Della Sala, Assunta Borzacchiello, Aniello Costantini, Luigi Paduano, Leide P. Cavalcanti, Fabiana Tescione, Brigida Silvestri, Giuseppina Luciani

**Affiliations:** 1 Department of Chemical, Materials and Production Engineering, University of Naples Federico II, Piazzale Tecchio 80, 80125 Naples, Italy; 2 Institute of Polymers, Composites and Biomaterials, National Research Council, (IPCB-CNR), 80125 Naples, Italy; 3 Department of Chemical Sciences, University of Naples Federico II, Via Cinthia, 80126 Naples, Italy; 4 ISIS Neutron Source, STFC, Didcot OX11 0QX, UK; 5 Department of Civil, Architectural and Environmental Engineering, University of Naples Federico II, Via Claudio 21, 80125 Naples, Italy

**Keywords:** hybrid nanoparticles, sol-gel synthesis, SiO_2_, heparin, drug release, biocompatibility

## Abstract

Heparin plays multiple biological roles depending on the availability of active sites strongly influenced by the conformation and the structure of polysaccharide chains. Combining different components at the molecular scale offers an extraordinary chance to easily tune the structural organization of heparin required for exploring new potential applications. In fact, the combination of different material types leads to challenges that cannot be achieved by each single component. In this study, hybrid heparin/silica nanoparticles were synthesized, and the role of silica as a templating agent for heparin supramolecular organization was investigated. The effect of synthesis parameters on particles compositions was deeply investigated by Fourier Transform Infrared Spectroscopy (FTIR) and Thermogravimetric Analysis (TGA). Transmission Electron Microscopy (TEM) reveals a different supramolecular organization of both components, leading to amazing organic-inorganic nanoparticles with different behavior in drug encapsulation and release. Furthermore, favorable biocompatibility for healthy human dermal fibroblasts (HDF) and tumor HS578T cells has been assessed, and a different biological behavior was observed, ascribed to different surface charge and morphology of synthesized nanoparticles.

## 1. Introduction

Heparin is a natural sulfated polysaccharide belonging to the family of glycosaminoglycans. The ability to enhance the rate at which antithrombin inhibits serine proteases in the blood coagulation cascade makes it widely used as an anticoagulant drug. In addition, heparin interacts with different protein groups and exhibits important physiological and pharmacological biological activities, ranging from anti-inflammatory, regulation of both cell growth and differentiation through interaction with different growth factors, and control of chemokine signaling [1,2,3,4,5,6,7,8]. Many of these biological activities are ascribed to the structural organization of heparin chains [9,10].

The conformation and the structure of polysaccharide chains have a strong influence on the size and availability of active sites, thus driving heparin bioactivity [11,12,13]. However, the high degree of heterogeneity in its monosaccharide sequence makes structural customizability very difficult. Thus, comprehensive knowledge of heparin structure/activity relationships remains a still unexplored field. To date, there are no effective strategies to easily tune the structural organization of heparin required for exploring new potential applications.

Hybrid organic-inorganic nanomaterials offer extraordinary promise because of their unique size-dependent properties. An impressive advantage of combining different components in a single hybrid nanostructure is the chance to tune both the structural and functional properties of the resulting system in the desired fashion. Usually, marrying features of single materials at the molecular scale generates a unique structure with synergistic and emergent capabilities. In addition, the combination of different material types leads to challenges that cannot be achieved by each single component [14,15].

Recent studies proved that inorganic phases were able to tune biopolymer supramolecular structures, boosting their intrinsic properties and driving their overall biological activity [16,17,18,19,20,21]. Notably, silica has been identified as the ideal support for this approach due to its strong hydrophilicity, acknowledged biocompatibility, as well as tunable size, shape, porosity, and surface [17,22]. It appears that the nature of the interface as well as of interaction and links between the organic and the inorganic component represent a key point for the design of new hybrids with tunable properties.

In this study, silica has been investigated as a templating agent for heparin supramolecular organization following the ceramic templated approach. Accordingly, hybrid heparin/silica nanoparticles (NPs) were synthetized through an in-situ wet chemistry approach. Furthermore, the effect of synthesis parameters on particle morphology and porosity was investigated by varying the initial amount of heparin to change the heparin to silica molar ratio. The hybrid NPs were tested for rhodamine-B-isothiocyanate encapsulation, evidencing different trapping, and releasing behavior, depending on their different structures. Overall, combining heparin with an inorganic structure proved to be an effective and straightforward way to tune the final hybrid structure as well as drug encapsulation and release features, providing the chance to make useful heparin organic–inorganic NPs via a simple synthesis strategy manipulation.

## 2. Materials and Methods

### 2.1. Materials

Heparin in the form of sodium salt (Hep, M_W_ = 13,870 Da) was kindly provided by LDO Company (Torino, Vercelli, Italy). 3-Aminopropyltriethoxysylane (APTS), N-(3-Dimethylaminopropyl)-N′-ethylcarbodiimide hydrochloride (EDC), tetrapropyl orthosilicate (TPOS), isopropanol, ammonium hydroxide, and rhodamine-B-isothiocyanate (RBiso) were purchased from Sigma-Aldrich (Milan, Italy) and used as received.

### 2.2. Synthesis of SiO_2_ and Heparin-SiO_2_ NPs

Heparin-SiO_2_ NPs were prepared by sol-gel synthesis as previously described [23] through a chemical conjugation between the carboxyl groups of heparin with amino groups of APTS.

Briefly, appropriate heparin amounts were used to produce hybrid nanoparticles with different APTS/-COOH molar ratios. Heparin is built up of linear chains of repeating disaccharide units (consisting of glucosamine and uronic acid) with a single carboxyl group; the amount of these groups was estimated through the ratio of the molecular weight of heparin to the disaccharide unit one. Table 1 reports the sample name, APTS/-COOH molar ratio, and heparin nominal amount (mg) of all synthesized samples.

In the general procedure, carboxyl groups of heparin were first activated in water with EDC and conjugated to amino groups of APTS. The crosslinking reaction was allowed to proceed overnight. Then, ammonia, isopropanol, and TPOS were added dropwise in such amounts as to have final ammonia, water, and total alkoxides concentrations in isopropanol equal to 0.1, 17, and 0.17 M (TPOS/APTS = 50/1 mol/mol), respectively. The system was kept at room temperature under magnetic stirring for 3 h, and the particles were purified by centrifugation and washed thrice with water. SiO_2_ NPs were produced analogously but without the addition of heparin [23].

### 2.3. Characterization Techniques

Morphological analysis of the prepared nanoparticles was carried out by Transmission Electron Microscopy (TEM). Samples were prepared by dispersing the obtained powders in an aqueous solution and then placing a drop of suspension on one side of the transparent polymer-coated 200 mesh copper grid. TEM images have been taken by a PHILIPS EM208S microscope equipped with a Mega View camera for image digital acquisition. Energy dispersive X-ray spectroscopy (EDX) investigation was carried out by pouring a drop of NPs suspension onto the surface of an aluminum stub, followed by sputter-coating with a Pt/Pd layer (5 nm) in a Cressington sputter coater 208HR. Then, the samples were analyzed by using a Field Emission Ultrapluss ZEISS Microscope.

N_2_ physisorption experiments were carried out to determine the specific surface area and the pore structure of hybrid NPs. Samples were ground into fine powders and degassed for 10 h at 0.03 mbar and 100 °C before the collection of adsorption/desorption isotherms by using a Micromeritics TriFlex instrument (Micromeritics, Norcross, GA, USA). Specific surface area (SSA) was evaluated by Brunauer Emmett Teller (BET) method, whereas the application of the Barrett Joyner Halender (BJH) model was applied on the adsorption branch of the isotherm to calculate pore size distributions and pore diameter.

Thermogravimetric analysis was performed in a simultaneous thermoanalyser SDT Q600 (TA Instrument, New Castle, DE, USA) at a heating rate of 10 °C/min under a nitrogen atmosphere. All experiments were carried out in triplicates to assess the reproducibility of the proposed synthesis. Considering the residual mass in the hybrid systems at 970 °C as the contribution of both components, from the equation:

$$W_{SiO_{2-HEP}}^{970{}^{\circ}C}=W_{SiO_{2}}^{970{}^{\circ}C}\cdot SiO_{2}\;wt\%+W_{Hep}^{970{}^{\circ}C}\cdot Hep\;wt\%$$

heparin weight percentage (*Hep wt%*) was derived as follows:

$$Hep\;wt\%=\frac{W_{SiO_{2}}^{970{}^{\circ}C}-W_{SiO_{2-HEP}}^{970{}^{\circ}C}}{W_{SiO_{2}}^{970{}^{\circ}C}-W_{Hep}^{970{}^{\circ}C}}\cdot 100$$

where $\;W_{SiO_{2-HEP}}^{970{}^{\circ}C}$ is the residual weight of synthesized hybrid particles, while $W_{SiO_{2}}^{970{}^{\circ}C}$ and $W_{Hep}^{970{}^{\circ}C}$ are the residual weight of bare silica and bare heparin, evaluated on dry basis (net of the initial water amount).

Fourier-transform infrared (FTIR) transmittance spectra were recorded in 400–4000 cm^−1^ using a Nicolet instrument (Nexus model) equipped with a DTGS KBr (deuterated triglycinesulfate with potassium bromide windows) detector. Powders of the samples were finely ground and pressed into pellets 2 cm in diameter. Potassium bromide was used as a background for spectrum collection.

Size distribution and ζ-potential measurements were carried out using Zetasizer Nano Series (Malvern Instruments, Malvern, UK), using the laser dynamic scattering (λ = 632.8 nm) and the particle electrophoresis techniques. All the samples were diluted in distilled water, and five runs (lasting 100 s) were used with a detecting angle of 173 in the calculations of the particle diameter distribution. The ζ-potential measurements were carried out by setting 50 runs for each measurement.

Small Angle Neutron Scattering (SANS) measurements were carried out on the Sans2d beamline at the ISIS Pulsed Neutron Source (STFC Rutherford Appleton Laboratory, Didcot, UK). A simultaneous Q-range of 0.004–1 Å^−1^ was achieved utilizing an incident wavelength range of 1.75−16.5- Å in time of flight mode and employing an instrument set up of L1 = L2 = 4 m and two detectors to measure small and wide angles, with the front detector at 2.4 m from the sample. Samples were prepared in deuterated water, providing the necessary contrast, and were contained in 2 mm path length Hellma quartz cells. Mantid Project was used to reduce data [24]. Each raw scattering data set was corrected for the detector efficiencies, sample transmission, and background scattering and converted to scattering intensity I(q) versus the scattering vector q = 4πn sin (θ/2)/λ, where λ and θ represented the wavelength of the neutron beam and scattering angle, respectively. The obtained profiles were fitted by using the SASwiew program (http://www.sasview.org/, accessed on 28 July 2022) to extrapolate the structural information about the hybrid nanoparticles.

Drug loading and release behavior were analyzed through UV-vis absorption measurements using a SHIMADZU UV-2600i (Shimadzu, Milan, Italy) spectrometer.

### 2.4. Drug Loading and Release Properties

#### 2.4.1. Adsorption Capacity

First, 500 μg of each type of NPs were suspended into 5 mL of phosphate buffer solution (PBS) to have three 100 μg/mL buffer suspensions. Then, 100 μg of RBiso were dissolved into each batch. All the suspensions were kept under mild stirring for 2 h. Afterward, NPs were centrifugated (12,000× *g* rpm, 10 min), and the supernatants were submitted to absorption measurement at λ = 544 nm to detect the dye concentration at the end of the adsorption time. The concentration value was then multiplied for the volume of the batch to calculate the dye weight in the solution. Finally, the weight of the adsorbed dye was determined by subtraction. The dye loading amount was evaluated as the micrograms of absorbed dye per gram of NPs, while the encapsulation efficiency (*EE*) for each sample was expressed as follows:

$$EE\left(\%\right)=\frac{W_{0}-W_{f}}{W_{0}}\cdot 100$$

where *W_0_* and *W_f_* are the weights of the dissolved dye at the beginning and the end of the adsorption process, respectively.

#### 2.4.2. Release Study

Dye-loaded nanoparticles were suspended in 5 mL of PBS and kept in a thermostatically controlled water bath. An amount of 600 μL of the suspension were withdrawn at fixed times (15′, 30′, 60′, 90′, 120′, and 24 h), centrifugated (12,000 rpm, 10′), and the supernatants were analyzed at UV-vis to determine the concentration, as previously specified. After every withdrawal, the suspensions were filled with an equal volume of fresh PBS. The release profiles are reported in terms of dye release (*DR*%) over time, where dye release is expressed as:

$$DR\left(\%\right)=\frac{C_{t}}{C_{\infty }}\cdot 100$$

where *C_t_* and *C_∞_* are the dye concentration at time *t* and if it would diffuse completely in the PBS suspension, respectively.

### 2.5. Biocompatibility Study

#### 2.5.1. Cell Culture

In order to test the biological response to NPs, Human Dermal Fibroblast (HDF) and Human Fibrosarcoma cell line (HT1080) were used as models of healthy and tumor tissue, respectively. The latter were cultured with complete medium composed of Eagle’s Minimum Essential Medium (EMEM) supplemented with 10% fetal bovine serum (FBS, Gibco), 100 U/mL penicillin, 100 mg mL^−1^ streptomycin. HDF cells were grown with EMEM supplemented with 20% FBS, 100 U/mL penicillin, 100 mg mL^−1^ streptomycin, and 2× non-essential amino acids. The cells were maintained in a humidified, controlled atmosphere with a 95% to 5% ratio of air/CO_2_ at 37 °C. The medium was changed every 3–4 days.

#### 2.5.2. Cell Viability Test

To understand the cell viability, NPs formulations SiO_2_-Hep_1, SiO_2_-Hep_3, and SiO_2_-Hep_5 were exposed to HDF cells and HS578T at a concentration of 10, 30, and 50 μg/mL, respectively. HDF cells were seeded at a density of 10 × 10^3^ cells/mL on 96-wells (World Precision Instruments, Inc.). The cells were seeded for each well in triplicate, cultured for 24 and 48 h, then Alamar blue assay (AB) was performed by adding AB reagent to the samples (at 10% *v*/*v* with respect to the medium) and incubated at 37 °C for 4 h. The absorbance of the samples was measured using a spectrophotometer plate reader (Multilabel Counter, 1420 Victor, Perkin Elmer) at 570 nm and 600 nm. AB is an indicator dye that incorporates an oxidation-reduction indicator that changes color in response to the chemical reduction in the growth medium, resulting from cell viability. Data are expressed as the percentage difference between treated and control to evaluate the percentage of reduction (*Reduction* %), which is calculated with the following formula:

$$Reduction\;\left(\%\right)=\frac{\left(O_{2}\cdot A_{1}\right)-\left(O_{1}\cdot A_{2}\right)}{\left(O_{2}\cdot P_{1}\right)-\left(O_{1}\cdot P_{2}\right)}\cdot 100$$

where 𝑂_1_ is the molar extinction coefficient (𝐸) of oxidized AB at 570 nm; 𝑂_2_ is the 𝐸 of oxidized AB at 600 nm; 𝐴_1_ is the absorbance of test wells at 570 nm; 𝐴_2_ is the absorbance of test wells at 600 nm; 𝑃_1_ is the absorbance of control well at 570 nm; and 𝑃_2_ is the absorbance of control well at 600 nm. The percentage of reduction for each sample was normalized to the percentage of reduction for the control to obtain the cell viability percentage [25].

#### 2.5.3. Confocal Microscopy for Cytoskeleton Staining

For cell morphology assay, cells were seeded at a density of 1 × 10^4^ cells/mL on fluorodish −35 mm (World Precision Instruments, Inc.), and 30 μg of the NPs formulations SiO_2_-Hep_1, SiO_2_-Hep_3, and SiO_2_-Hep_5 were incubated for 24 h. Then, samples were washed two times with PBS and fixed with 10% formaldehyde for 1 h at 4 °C. The fixed cells were permeabilized with Triton X-100 0.1% in Phosphate-buffered saline (PBS) for 3–5 min. The actin filaments were stained with FITC phalloidin (Cayman Chemical Company, Ann Arbor, MI, USA) in PBS for 30 min at room temperature. Finally, after two washes with PBS to remove unbound phalloidin conjugate, cell nuclei were stained with 4′,6-diamidino-2-phenylindole, DAPI, (Sigma-Aldrich, Milan, Italy). The samples were observed by a confocal microscope system (Leica TCS SP8) with a 63× oil immersion objective. Images were acquired with a resolution of 1024 × 1024 pixel.

## 3. Results and Discussion

Coupling organic components to inorganic precursors via the wet chemistry route is frequently used as it represents a simple and effective process for tuning sophisticated hybrid structures, preserving the organic phase in the final architecture.

**Figure 1:**
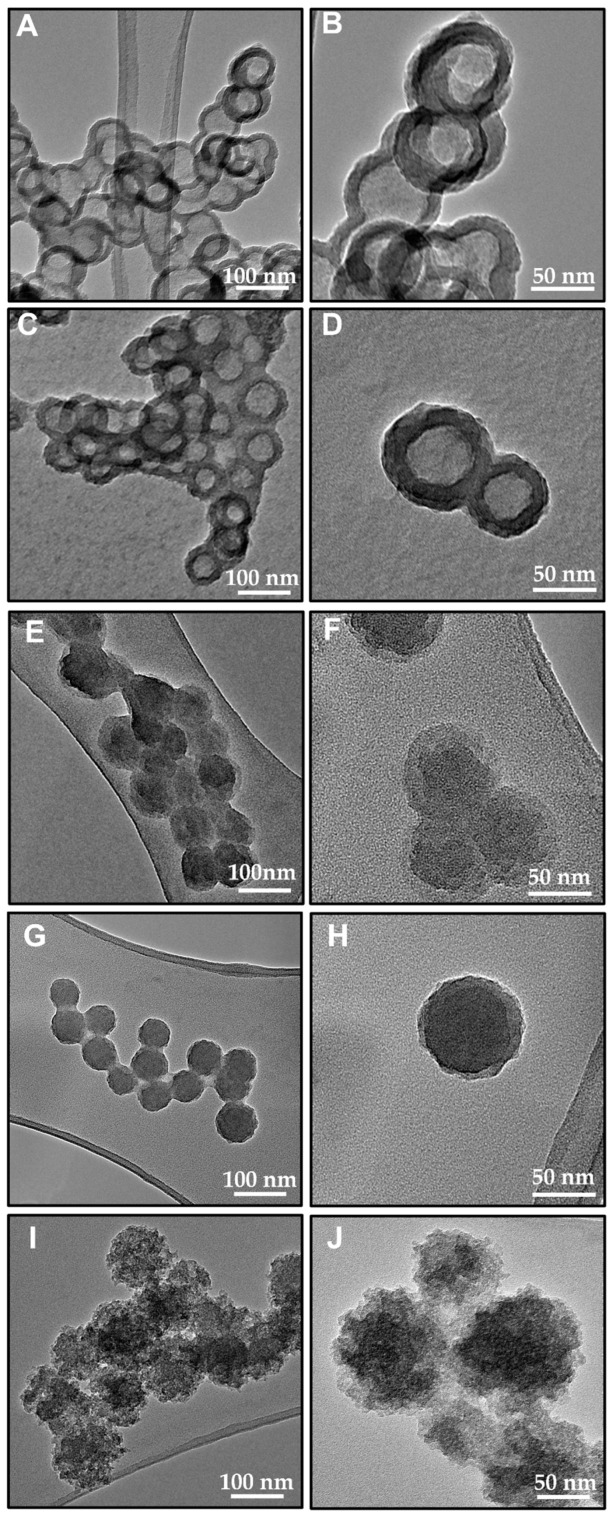
TEM micrographs of SiO_2_-Hep_1 (**A**,**B**), SiO_2_-Hep_2 (**C**,**D**), SiO_2_-Hep_3 (**E**,**F**). SiO_2_-Hep_4 (**G**,**H**) and SiO_2_-Hep_5 (**I**,**J**) nanoparticles, taken at lower (left column) and higher (right column) magnification.

In this work, heparin-silica-based hybrids were prepared at room temperature using APTS as a coupling agent between the two phases. Figure 1 reports TEM images of hybrid particles synthesized using different heparin amounts.

All synthesized samples showed a pseudospherical shape and a size of about 90 nm in diameter. In the micrographs of both SiO_2_-Hep_1 (Figure 1A,B) and SiO_2_-Hep_2 (Figure 1C,D) samples, where a lower amount of heparin was used, core-shell structures were clearly visible by the difference in contrast between the outer darker layer about 20 nm thick and the lighter inner core. As the amount of heparin in the starting solution increased, the structure of the NPs evolved into more uniform architectures (Figure 1E–H) where darker inner cores were surrounded by a lighter halo, about 10 nm in thickness. On the other hand, at higher heparin concentration (SiO_2_-Hep_5 NPs) the core-shell structure was no longer visible, and the resulting structure (Figure 1I,J) appeared quite different from all other systems, showing a ‘cloudy’ structure with no well-defined shape [26].

**Figure 2:**
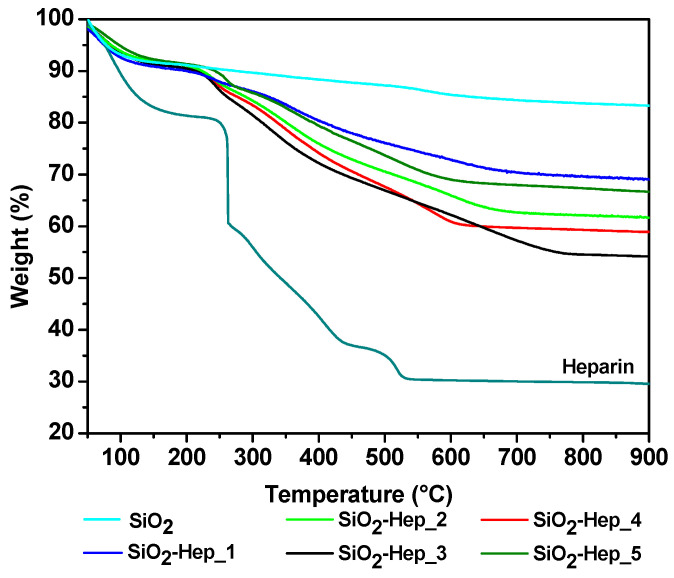
TGA thermograms of bare Heparin, SiO_2_, and SiO_2_-Hep hybrid nanoparticles.

In order to assess the amount of heparin loaded into the silica skeleton, TGA measurements were performed on all hybrid systems, and the results were compared with those of neat heparin and bare SiO_2_ NPs. Figure 2 reports the TGA curves of all samples, whereas the actual amount of the organic phase in the final hybrid systems, calculated as described in the experimental section, was reported in Table 2.

All TGA curves (Figure 2) exhibited a first weight loss at temperatures lower than 150 °C, attributed to the loss of adsorbed water. In the thermogravimetric curve of the silica-gel nanoparticles, a very small weight change of about 3 wt% was observed at higher temperatures, usually associated with the decomposition of residual alkoxides groups and/or dehydroxylation [27]. The TG curve of neat heparin showed a pronounced weight loss in the region between 200–600 °C due to the heparin thermal decomposition and a residual mass of about 30 wt% at 900 °C [23,28].

Compared with bare SiO_2_ samples, all hybrid NPs showed larger weight loss above 200 °C due to the degradation of the organic phase, suggesting the presence of heparin. Notably, the SiO_2_-Hep_3 sample showed the greatest loss above 200 °C, indicating the highest heparin content (53%); in addition, the recorded mass loss was distributed in a higher temperature range, suggesting better thermal stability of heparin. This result suggests that the conjugation of APTS and heparin is more effective in the SiO_2_-Hep_3 samples, which not only show the largest heparin amount but also the highest thermal stability among all investigated samples. On the other hand, in the SiO_2_-Hep_5 samples, even though the highest organic content used during the synthesis influenced particle morphology (Figure 1I,J), the excess heparin was certainly removed during the particle washing procedure, producing a final sample that contained a heparin content comparable to SiO_2_-Hep_1 sample (31% vs. 25%, respectively), which was prepared to start from a far smaller heparin amount.

**Figure 3:**
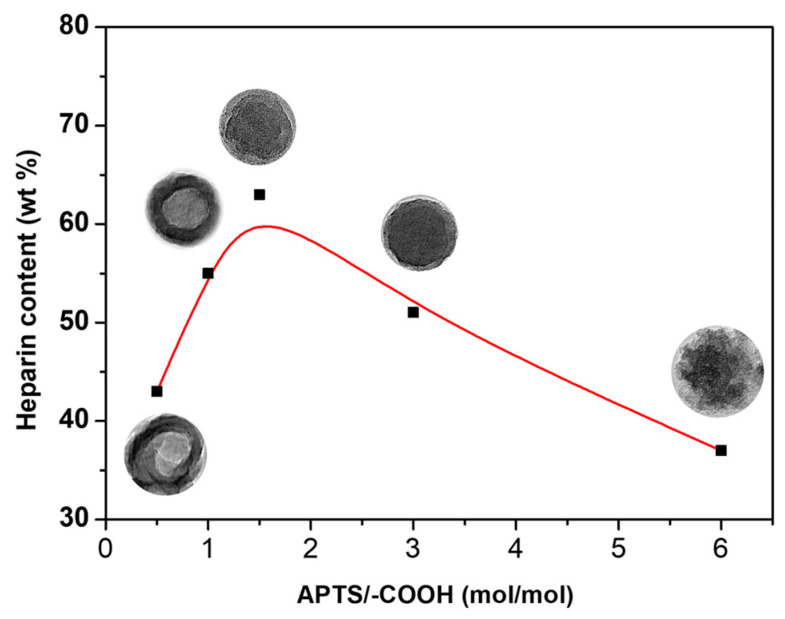
Heparin content vs APTS/-COOH molar ratio with corresponding morphological structure evolution.

Furthermore, Figure 3 shows heparin weight percent, as determined by TG curves, as a function of APTS/-COOH molar ratio.

Based on both morphological and compositional analysis, three regimes can be identified depending on heparin nominal content, which gives rise to three different NP architectures:

(I) Core-shell structure at low heparin amount, with inorganic component probably in the outer layer;
(II) Inverted core-shell structure for heparin amounts comparable to APTS, in which the shell might likely be made by heparin;
(III) Cloudy structure essentially made by heparin if its content is far larger than APTS.

Consequently, a more exhaustive investigation was carried out on the following samples: SiO_2_-Hep_1, SiO_2_-Hep_3, and SiO_2_-Hep_5 NPs, which are representative of the three regimes.

**Figure 4:**
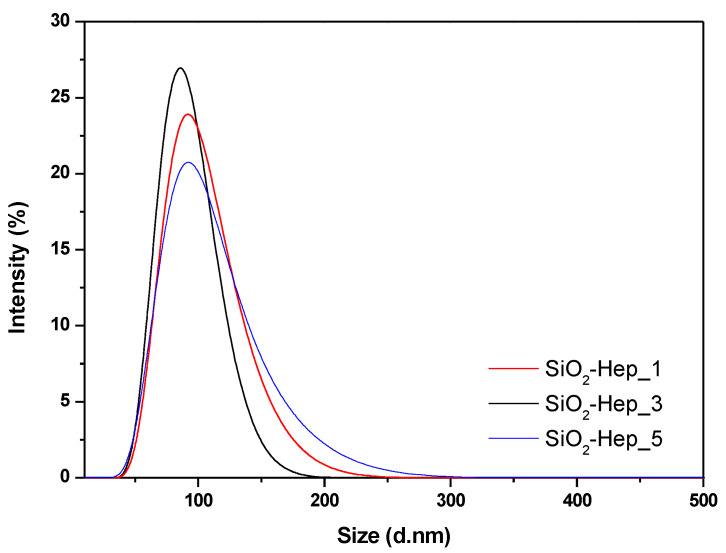
DLS number distributions of SiO_2_-Hep_1, SiO_2_-Hep_3, and SiO_2_-Hep_5 NPs.

The nanoparticles size distribution of these samples was investigated through DLS measurements reported in Figure 4.

DLS analysis showed a mean hydrodynamic diameter of about 90 nm in the case of the SiO_2_-Hep_3 sample. Much larger particle sizes, of about 100 nm, were observed for both SiO_2_-Hep_1 and SiO_2_-Hep_5 NPs. In addition, in agreement with the TEM investigation, this latter sample exhibited a wide size distribution, probably related to the presence of particles with different diameters.

**Figure 5:**
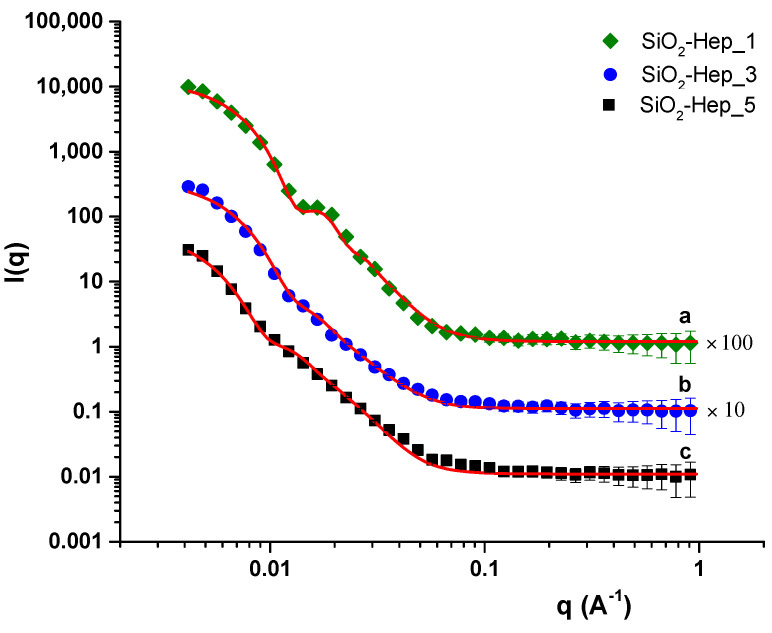
SANS profiles and corresponding best fitting curves of SiO_2_–Hep_1 (**a**), SiO_2_–Hep_3; (**b**) and SiO_2_–Hep_5; (**c**) suspensions in D_2_O.

SANS measurements were performed to investigate the structural organization of SiO_2_-Hep nanohybrids. All the suspensions were prepared in D_2_O to exploit the difference in scattering length density between the solvent (*ρ*_D_2_O_ = 6.34 × 10^−6^ Å^−2^) and the nanostructures (*ρ*_SiO_2__ = 3.4 ×10^−6^ Å^−2^). Three specific compositions were considered, falling in each of the three regimes, which were previously identified: SiO_2_-Hep_1, SiO_2_-Hep_3, and SiO_2_-Hep_5. Figure 5 shows the collected experimental data reported together with the corresponding fitting curves for all the analyzed systems.

The quantitative analysis of SANS data was based on the identification of the suitable model to describe the scattering intensity, *I(q)*, versus the scattering vector, *q*, in order to extract structural information of SiO_2_-Hep nanohybrids. Specifically, although some differences were observed in the supramolecular organization of the three samples in an aqueous environment, the core-shell sphere model was the best one to fit all SANS profiles. This provided the form factor, *P(q)*, for a spherical particle with a core-shell structure, which was normalized by the particle volume. The 1D scattering intensity from core-shell particles is calculated in the following way [29]:

$$P(q)=\frac{scale}{V}F^{2}(q)+bkg$$

where

$$F(q)=\frac{3}{V_{s}}[V_{c}(\rho_{c}-\rho_{s})\frac{\sin(qr_{c})-qr_{c}\cos(qr_{c})}{{\left(qr_{c}\right)}^{3}}+V_{s}(\rho_{s}-\rho_{solv})\frac{\sin(qr_{s})-qr_{s}\cos(qr_{s})}{{\left(qr_{s}\right)}^{3}}$$

where *V_s_* is the volume of the whole particle, *V_c_* is the volume of the core, *r_s_ = radius + thickness* is the radius of the particle, *r_c_* is the radius of the core, *ρ_c_* is the scattering length density of the core, *ρ_s_* is the scattering length density of the shell, *ρ_solv_* is the scattering length density of the solvent. Thus, the optimized values of these parameters were obtained from the best fitting of the experimental curves, as summarized in Table 3.

In all suspensions, the presence of core-shell nanostructures with mean diameters ranging between 40 and 60 nm as a function of heparin content was revealed (see Table 1). Moreover, a quite high polydispersity (between 0.3 and 0.7) of radius and thickness values was observed, independently by the heparin content used for the synthesis, thus confirming the presence of particles with different sizes, as already indicated by broad DLS distribution curves. At low heparin content (SiO_2_-Hep_1), the presence of nanostructures with an inner core of ~38 nm, mainly composed of SiO_2_ (*ρ ^theoric^* = 3.4 × 10^−6^ Å^−2^), and an outer one of ~6.5 nm was observed. This organization differed from that observed in TEM images, and it should be explained because of the very low colloidal stability and high polydispersity of this system. In fact, the suspension showed a great precipitation tendency, which mainly involved the prevailing population of particles that exposed the inorganic phase as the outer layer to water. These had a major propensity to self-aggregate and precipitate rapidly, as also suggested by the low zeta potential values lower than +30 mV. On the other hand, the population of SiO_2_ particles dispersed in the heparin network had a time-longer stability in aqueous suspension to be detected as prevalent ones in solution. At the higher heparin content (SiO_2_-Hep_3), the values of the fitting parameters agreed with the formation of hybrid nanostructures of about 48 nm composed of a SiO_2_-based core (~43 nm in diameter), which were coated with a very thin organic layer (~5 nm) of heparin exposed to the aqueous environment, in agreement with TEM. Finally, a particular situation was observed for the SiO_2_-Hep_5 sample. Indeed, the best fitting model suggested the presence of a core with a diameter of ~50 nm and of a thin shell of ~5 nm; however, the experimental *ρ* values resulted very similar between them and tended to that of D_2_O, suggesting that the composition of these “two layers” was not well-defined and presented a high hydration level, which should be compatible with the hybrid cloudy structure indicated by TEM.

**Figure 6:**
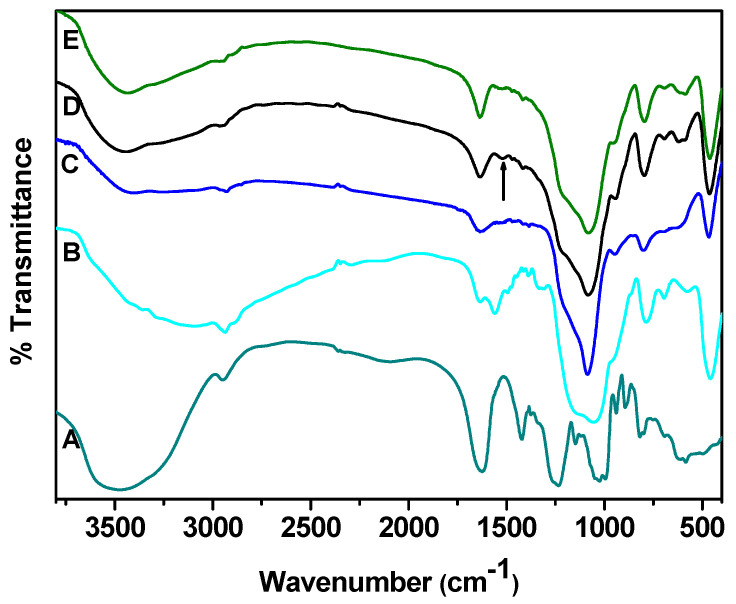
FTIR spectra of both bare heparin (**A**), silica (**B**), SiO_2_−Hep_1 (**C**), SiO_2_−Hep_3 (**D**), and SiO_2_−Hep_5 (**E**) hybrid nanoparticles.

Figure 6 shows the FT-IR spectra of bare heparin and silica NPs (a) as well as hybrid nanoparticles. The assignment of FT-IR bands is reported in Table 4.

**Scheme 1:**
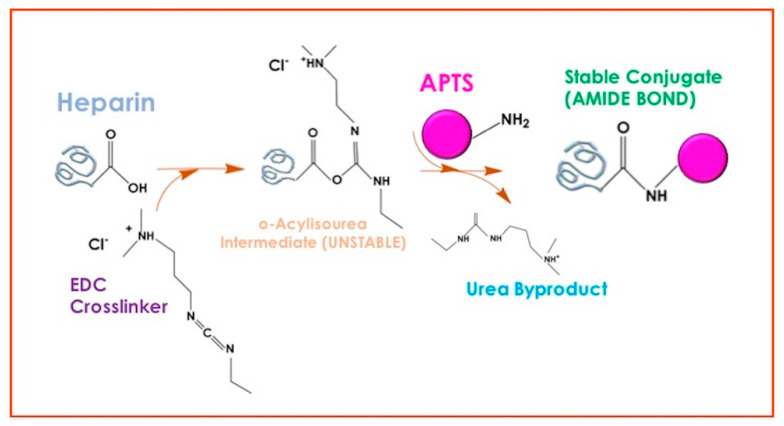
Schematic illustration of amide bond formation between amino groups of APTS and carboxyl groups of heparin.

FT-IR spectrum of SiO_2_ NPs exhibits the typical band of silica gel phase [26]. In addition, further weak bands can be appreciated at 2940 cm^−1^ and 2880 cm^−1^, which can be assigned to the stretching mode of the CH_2_ groups, whereas the absorption band at 1559 cm^−1^ is attributed to the bending mode of the -NH_2_ groups, both associated with the presence of APTS skeleton. All the spectra of hybrid NPs are dominated by the typical silica gel signals. Additionally, the adsorption band at 1560 cm^−1^ is no longer evident, whereas a new band at 1520 cm^−1^ is clearly visible and more pronounced in the SiO_2_-Hep_3 spectrum. Since it is related to stretching vibrations of N–H and C–H in amide II bond formation, it confirms chemical coupling between the two phases. A schematic illustration of the reaction mechanism is reported in Scheme 1.

Furthermore, as heparin content increases, the band at about 1080 cm^−1^, assigned to Si–O–Si stretching vibration in SiO_4_ units (Table 4), shifts toward lower wavenumbers, thus suggesting that the higher heparin content in synthesis results in a less crosslinked silica network [21,30,31,32].

Additional textural and chemical characterizations were carried out on hybrid samples. Table 5 reports surface charge (ζ-Pot), SSA, and the elemental EDX analysis related to bare silica and hybrid NPs.

Pure silica nanoparticles showed a positive surface charge attributed to the presence of protonated amino groups (-NH_3_^+^) of silica particles. As reported in Table 5, the SiO_2_-Hep_1 sample showed a positive charge, while both SiO_2_-Hep_3 SiO_2_-Hep_5 samples showed a negative surface charge. These results suggested the presence of an outer surface layer composed of silica in the SiO_2_-Hep_1 sample and confirmed the low colloidal stability which favored the precipitation, as suggested by the analysis of SANS data, whereas in both SiO_2_-Hep_3 and SiO_2_-Hep_5 samples, heparin component was mainly exposed on the surface of the nanoparticles [11,33,34].

Additionally, EDX analysis showed a significant amount of C, N, and S elements in both SiO_2_-Hep_3 and SiO_2_-Hep_5 samples, suggesting a higher exposure of heparin component to the NPs. These elements cannot be detected on the surface of the SiO_2_-Hep_1 sample (except for 6%mol carbon). Furthermore, such high Si content confirms the presence of a silica surface layer and a lower heparin content for this sample.

In order to investigate the influence of heparin on both surface area and porosity of hybrid NPs, N_2_ adsorption/desorption analysis was performed on all synthesized systems. Both bare SiO_2_ and SiO_2_-Hep_1 NPs showed very low surface area, as reported for ‘dense’ silica nanoparticles produced through the Stöber method [35,36].

A slightly high surface area was obtained for SiO_2_-Hep_3, while a significant increase was appreciated in the case of the sample synthesized with the highest content of heparin, i.e., SiO_2_. Both ζ-Pot and SSA values confirm the possibility of easily tuning porosity and surface features by varying the amount of organic component during the synthesis and, at the same time, the active role of the same component in modulating the morphology, the structure, and the porosity of the final nanoparticles [25,37].

Based on obtained results, a possible mechanism can be depicted in order to explain the role of the different amounts of heparin in defining the final nanoparticle architectures.

On the basis of the nucleation theory, in the early stages of NP production, primary particles of about 5 nm in diameter are formed by the condensation reaction of hydrated monomers [38]. Then, primary particles aggregate, producing larger SiO_2_ particles that grow up to a stationary critical size, able to generate a double electrical layer that hinders any further aggregation [39,40,41].

The conjugation reaction performed at low heparin content (SiO_2_-Hep_1) resulted in the highest APTS/-COOH molar ratio. In this case, the presence of both ‘free’ and ‘bound’ APTS moieties are expected in the starting solution. The prehydrolysis of these APTS species is supposed to form a certain number of nuclei, and the ‘free’ APTS nuclei might reasonably condense on ‘heparin bound’ ones during the first overnight step. These preformed heterostructures (made of heparin-silica phases) can act as nucleation centers driving the further condensation process of hydrolyzed TPOS moieties.

**Scheme 2:**
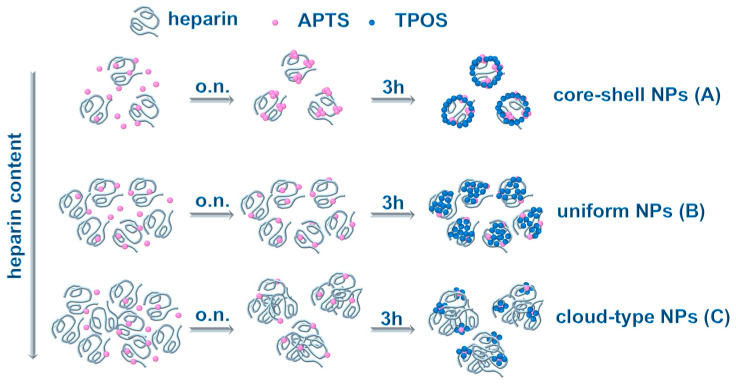
Formation mechanism of hybrid SiO_2_-Hep NPs by varying heparin content.

The proposed formation mechanism was reported in Scheme 2A: firstly, APTS molecules covalently bind to the activated carboxyl group of heparin. At the same time, ‘free’ hydrolyzed APTS moieties are formed, which might condense with OH groups produced by the hydrolysis of APTS-Heparin moieties. The local high concentration of anchored silica domains leads to the formation of a small number of heparin-silica nucleation centers and could feed the growth of particles. The condensation of TPOS hydrolyzed moieties become preferentially located at the surface of heparin-APTS nucleation centers, and the coalescence of branched oligomeric structure results in a silica shell formation. Therefore, the final hybrid particles exhibit a core-shell architecture made of a heparin-rich core surrounded by a silica shell.

At intermediate heparin content, Scheme 2B, APTS/-COOH molar ratio is almost 1, and only ‘bound’ APTS moieties are expected in the starting solution. In this case, when TPOS was added to a solution containing many heparin-APTS nucleation centers, the increased number of binding sites for hydrolyzed TPOS moieties led to a more homogeneous population of primary particles made of silica nuclei distributed onto heparin chains. In this case, the particles keep growing uniformly in diameter resulting in the final homogeneous size distribution of hybrid particles with heparin both inside and at the surface. More specifically, heparin chains located on the surface of the nanoparticles organize themselves into a thin shell, as revealed by TEM measurements.

The increase in the heparin amount reduces the rate of silica phase polycondensation reaction due to the steric hindrance effect. The organic phase acts as a vessel for primary particle formation, but, at the same time, it hinders their further aggregation by acting as a barrier to the penetration of silica particles in the aggregation step (Scheme 2C). The resulting cloud-type morphologies were probably made of small inorganic silica domains surrounded by cross-linked heparin chains.

SiO_2_-Hep_1, SiO_2_-Hep_3, and SiO_2_-Hep_5 samples, representative of each different morphology, were then tested in the drug loading amount and encapsulation efficiency of RBiso as a drug model. Table 6 showed that RBiso loading amount was about 140 μg/mg in both SiO_2_-Hep_1 and SiO_2_-Hep_3 samples, while it decreased to 45 μg/mg in SiO_2_-Hep_5 NPs. This resulted in an encapsulation efficiency of ~70% in both SiO_2_-Hep_1 and SiO_2_-Hep_3 samples, whereas it was only 22.6% in SiO_2_-Hep_5.

Electrostatic attractions between the positive charge of the dye and the negative charge of heparin together with the hydrophobic interactions cationic dyes are easily adsorbed onto heparin-functionalized systems [42], resulting in the highest absorption efficiency obtained in the SiO_2_-Hep_3 sample. ζ-Pot measurements evidenced the presence of surface amine groups onto the SiO_2_-Hep_1 sample. These moieties might be involved in the nucleophilic addition reaction with the isothiocyanate groups of the dye, thus justifying the high adsorption efficiency also detected in this case. On the contrary, in the SiO_2_-Hep_5 sample, the drug loading amount achieved the lowest values. Despite the largest specific surface area among the investigated samples, the lower heparin content than the SiO_2_-Hep_3 sample negatively affected the encapsulation efficiency of RBiso.

**Figure 7:**
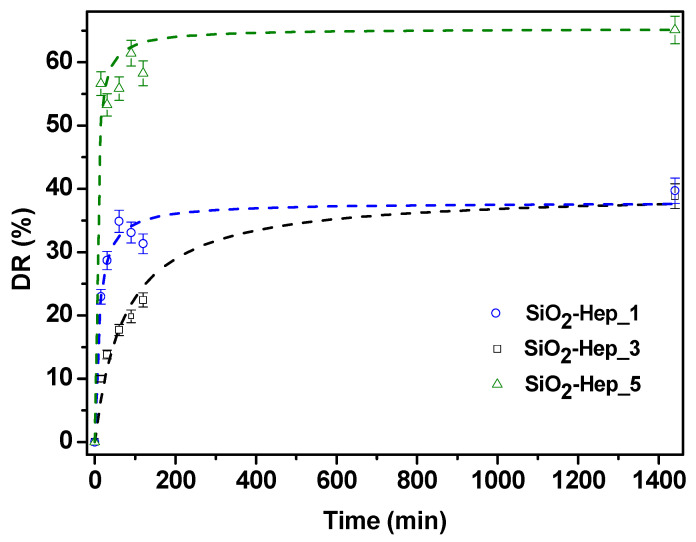
Release profiles of RBiso from SiO_2_-Hep_1, SiO_2_-Hep_3, and SiO_2_-Hep_5 samples.

The RBiso release profiles were reported in Figure 7, which clearly showed that the different architectures of hybrid NPs strongly affected the release behavior. The desorption process of the dye from both SiO_2_-Hep_1 and SiO_2_-Hep_5 samples was faster than the case of SiO_2_-Hep_3, showing an initial burst release that reached a constant value within only 4h. Furthermore, the SiO_2_-Hep_5 sample released almost 65% of encapsulated dye, while in the case of SiO_2_-Hep_1, a final released amount of 39% was observed. Finally, a smoother dye release was observed in the SiO_2_-Hep_3 NPs. This might likely be ascribed to the highest heparin content as well as to its more uniform distribution within the sample, as supported by morphological analysis.

**Figure 8:**
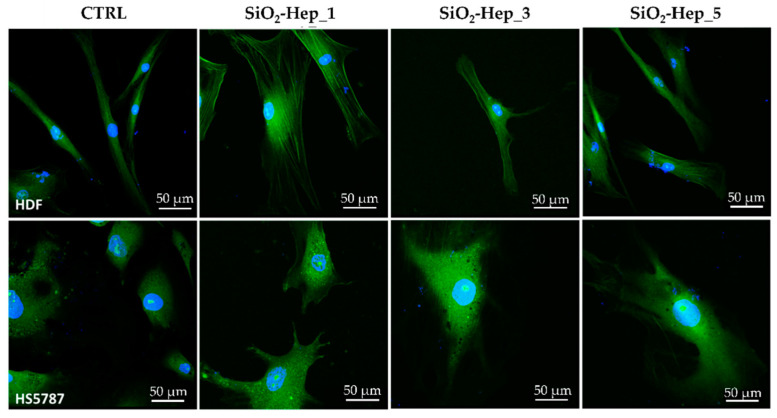
Cell morphology for the control cells and cells incubated with SiO_2_-Hep_1, SiO_2_-Hep_3, SiO_2_-Hep_5 NPs, at concentration of 30 µg, respectively, after 24 h. In Green Actin filaments stained by phalloidin-FITC and in Blue DAPI stained nuclei cells. All bars represent 50 μm.

The biocompatibility of the SiO_2_-Hep_1, SiO_2_-Hep_3, and SiO_2_-Hep_5 hybrid NPs was firstly confirmed by cells morphology evaluation. Actin filaments, a constituent of the cytoskeleton, were stained with FTIC phalloidin after 24 h of incubation with the different NP formulations. Both healthy HDF cells and HS578T cancer cells (Figure 8) exhibited a no cytotoxicity morphology characterized by a spread or spindle-shaped appearance and the presence of several extending processes, showing cells protrusion adhering to the flat surface.

**Figure 9:**
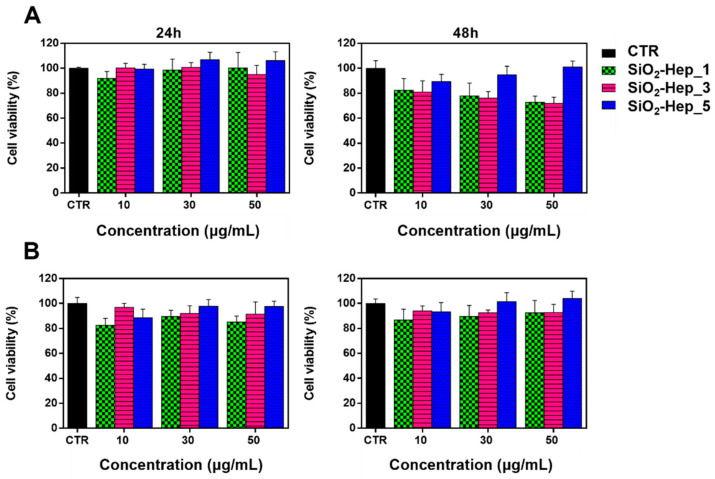
Cell Viability percentage of (**A**) HDF and (**B**) HS578T cells lines after 24 and 48 h of incubations with SiO_2_-Hep_1, SiO_2_-Hep_3, and SiO_2_-Hep_5 NPs, at concentration of 10, 30, and 50 µg.

In order to evaluate the biocompatibility of SiO_2_-Hep_1, SiO_2_-Hep_3, and SiO_2_-Hep_5 hybrid NPs, the viability of healthy HDF and tumor HS578T cells has been assessed by the Alamar Blue assay after 24 and 48 h of incubation. The three selected species of NPs exhibited favorable biocompatibility for both cells line. The cell viability percentage does not drop for any formulation below 80% of cell viability, indicating in accordance with the ISO 10993–5: 2009 [43] that NPs are well tolerated in cellular applications. As shown in Figure 9A, after 24 h of incubation at all the tested concentrations (10, 30, and 50 µg/mL), the viability of the HDF cells was about 100%, while after 48 h a slight decrement of the viability rate has been estimated. In particular, for the SiO_2_-Hep_1, SiO_2_-Hep_3 NPs, a decrease in cell viability was noticed when the concentration of NPs increased, and vice versa for the SiO_2_-Hep_5 formulations, an increase in viability was found as the concentration of NPs increased. Overall, these results proved the safety of the three formulations. However, SiO_2_-Hep_5 demonstrated better biocompatibility after 48 h compared to SiO_2_-Hep_1, SiO_2_-Hep_3 NPs. This biological behavior can be ascribed to surface charge, morphology, and content of heparin in the different formulations. In particular, NPs with zeta potential values that fall between –30 mV and +30 mV indicate poor colloidal stability and greater ease of aggregation [44]. NPs aggregation phenomena might provoke cytotoxic effects by enhancing NP accumulation in certain body compartments or inside cells [45,46]. This would explain the slight decrease in viability at 48 h for the SiO_2_-Hep_1 and SiO_2_-Hep_3 NPs, with a zeta potential of +20 and −19 mV, respectively, and the better biocompatibility of the SiO_2_-Hep_5 formulation synthesized with the highest content of heparin and with a superficial charge of −33 mV. The samples also demonstrated biocompatibility in HS578T tumor cells, with a similar trend between the different formulations (Figure 9B). This behavior may derive from the different natures of cancer cells. Indeed, cancer cells are different from healthy cells in various ways and divide at an unregulated rate. Moreover, most NPs exhibit different endocytic mechanisms with extensive cytoskeletal deregulation that would make tumor cells more prone to indiscriminate and unselected internalization of nanomaterials [47,48].

## 4. Conclusions

This study reports the synthesis of hybrid heparin/silica nanoparticles using an in-situ wet chemistry approach. Silica proved to be an effective templating agent for heparin with the aim to finely tune its supramolecular organization, ultimately influencing its potential applications.

Morphological and compositional analysis revealed three different NP architectures, which can be obtained by tuning the ratio between the two components:

(I) Core-shell structure with inorganic component probably located in the outer layer;
(II) Inverted core-shell structure in which the shell might likely be made of heparin;
(III) Cloudy architectures are essentially made of heparin domains.

The samples representative of each morphology were tested in drug loading and release experiments, selecting RBiso as a model drug. Results highlighted that the different architectures exerted a strong influence on the behavior of each type of nanoparticle. Furthermore, different surface charge and morphology resulted in a different cell to nanoparticles interaction which ultimately led to better biocompatibility observed for cloudy type architectures.

## Figures and Tables

**Table 1:** APTS/-COOH group molar ratio and nominal amount of heparin used during synthesis.

Sample Name	APTS/-COOH (mol/mol)	Heparin Nominal Amount (mg)
SiO_2_-Hep_1	6	25.9
SiO_2_-Hep_2	3	51.7
SiO_2_-Hep_3	1.5	103.4
SiO_2_-Hep_4	1	155.1
SiO_2_-Hep_5	0.5	310.5

**Table 2:** Estimation of heparin weight percentage for all the hybrid samples.

Sample	Heparin (wt. %)
SiO_2_-Hep_1	25
SiO_2_-Hep_2	40
SiO_2_-Hep_3	53
SiO_2_-Hep_4	44
SiO_2_-Hep_5	31

**Table 3:** Properties of hybrid SiO_2_/Hep nanostructures obtained by the best fitting of SANS curves.

Sample	Sphere Core Radius R (Å)	Shell Thickness t (Å)	*ρ_c_^exp^ × 10^−6^* (Å^2^)	*ρ _s_^exp^ × 10^−6^* (Å^2^)
SiO_2_-Hep_1	190 ± 25	65 ± 8	3.2	0.9
SiO_2_-Hep_3	213 ± 10	48 ± 7	3.4	1.1
SiO_2_-Hep_5	270 ± 35	47 ± 5	5.1	4.7

**Table 4:** Main absorption bands and assignments in the FTIR spectra.

Frequency (cm^−1^)	Possible Assignments
3400	OH stretching vibration
2949	stretching vibration of C-H bonds
1623	asymmetric axial deformation of carboxylate anions
1421	symmetric axial deformation of carboxylate anions
1232	asymmetric stretching of SO_3_
1100	Si–O–Si stretching vibration in SiO_4_ units
1040	symmetric stretching of SO_3_
950	Si–O terminal non-bridging vibration
937	vibration of C–O–C glycosidic bond and C–O–S stretching
890	C–O–S stretching with some coupling component of C–O–S stretching
800–820	S–O–C stretching
470	Si–O–Si bending

**Table 5:** Zeta-Potential (ζ-Pot), SSA and EDX analysis.

Name	ζ-Pot	Si/O/C/N/S EDX (%mol)	SSA m^2^/g
SiO_2_	+15 ± 0.8	44/39/0/0/0	20
SiO_2_-Hep_1	+20 ± 1.1	40/50/6/0/0	26
SiO_2_-Hep_3	−19 ± 0.6	23/48/23/4/2	45
SiO_2_-Hep_5	−33 ± 0.9	30/56/10/3/2	102

**Table 6:** Encapsulation efficiency and drug release of RBiso. The reported values fall within the limit values for experimental error of 5%.

Sample	Dye Loading Amount (μg/mg)	Encapsulation Efficiency (%)	Drug Release (%)
SiO_2_-Hep_1	142.70	71.35	38.85
SiO_2_-Hep_3	145.85	72.93	39.71
SiO_2_-Hep_5	45.11	22.56	65.10
